# Anti-Inflammatory Effects of Total Isoflavones from *Pueraria lobata* on Cerebral Ischemia in Rats

**DOI:** 10.3390/molecules180910404

**Published:** 2013-08-28

**Authors:** Dong Wook Lim, Changho Lee, In-Ho Kim, Yun Tai Kim

**Affiliations:** Functionality Evaluation Research Group, Korea Food Research Institute, Seongnam 463-746, Korea

**Keywords:** isoflavones, *Pueraria lobata*, anti-inflammatory, cerebral ischemia

## Abstract

*Puerariae* radix, the dried root of *Pueraria lobata* Ohwi, is one of earliest and most important edible crude herbs used for various medical purposes in Oriental medicine. The aim of the present study was to determine the anti-inflammatory effects of Total Isoflavones from *P. lobata* (TIPL), which contains the unique isoflavone puerarin, in ischemia *in vivo* models. Oral administration of TIPL (100 mg/kg) reduced the brain infarct volume and attenuated ischemia-induced cyclooxygenase-2 (COX-2) up-regulation at 2 days after middle cerebral artery occlusion (MCAo) in rats. Moreover, TIPL reduced activation of glial fibrillary acid protein (GFAP) and CD11b antibody (OX-42) at 7 days after MCAo in hippocampal CA1 region. These results show that TIPL can protect the brain from ischemic damage after MCAo. Regarding the immunohistochemical study, the effects of TIPL may be attributable to its anti-inflammatory properties by the inhibition of COX-2 expression, astrocyte expression, and microglia.

## 1. Introduction

The main focus of drug development to protect ischemia-induced injury has been the investigation of neuroprotective sources capable of protecting neurons from ischemic cell death. Natural products, especially medicinal plants, could be an ideal source to develop safe and effective agents for neuroprotection against ischemia-induced injury [1].

*Puerariae* radix, the dried root of *Pueraria lobata* Ohwi, is one of earliest and most important edible crude herbs used for various medical purposes in Oriental medicine. It is commonly employed to relieve fever and dysentery, and for the treatment of cardiovascular diseases such as hypertension, myocardial infarction and arrhythmia. *Puerariae* radix has been reported to display anti-oxidant [2], hypoglycemic [3] and anti-thrombosis effects [4], as well as lowering plasma cholesterol [5]. The major active components of *Puerariae* radix are flavonoids, coumarins and isoflavones such as daidzein, genistein, puerarin that are presumed responsible for its diverse pharmacological activities [6]. Especially, the isoflavones exhibit a wide range of biological activities, with anti-inflammatory, anti-thrombotic, anti-hypertensive, anti-arrhythmic, spasmolytic, and cancer chemopreventive properties [7,8].

The aim of the present study was to determine the anti-inflammatory effects of Total Isoflavones from *P. lobata* (TIPL), which contains the unique isoflavone puerarin, on ischemia *in vivo* model. We used a middle cerebral artery occlusion (MCAo) rat model to evaluate the potential protective effects against focal cerebral ischemia [9]. This model mimics aspects of the pathophysiology and sensory motor dysfunction of stroke in humans [10]. The effect on brain infarct volume was measured by 2,3,5-triphenyltetrazolium chloride (TTC) staining. We observed the expression of glial fibrillary acid protein (GFAP), CD11b antibody (OX-42), and cyclooxygenase-2 (COX-2) by immunohistochemistry to find out the inhibitory effects on microglia activation, astrocyte activation and COX-2 up-regulation which are related to inflammation.

## 2. Results and Discussion

### 2.1. Compositional Analysis of Total Isoflavones from P. lobata Extracts

**Figure 1:**
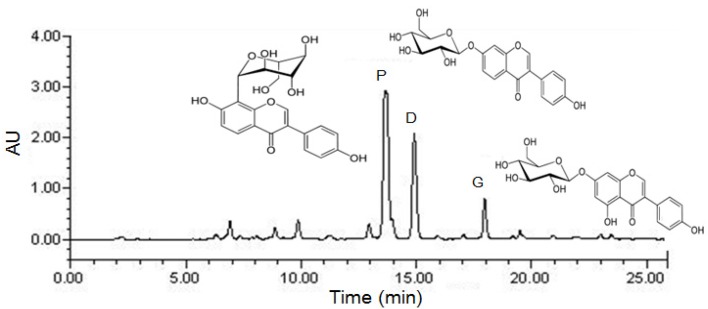
Preparative HPLC chromatography of Total Isoflavones from *P. lobata* (TIPL). P: puerarin; D: daidzin; G: genistin.

The HPLC chromatogram of total isoflavones is shown in Figure 1, and the puerarine, daidzin and genistin concentrations in the *P. lobata* extracts are listed in Table 1. After purification, puerarin (7.2%) was the major compound in the extract, which also contained daidzin (3.8%) and genistin (1.5%). The TIPL for oral administration was calculated based on its isoflavone contents.

**Table 1:** Composition of puerarin and other isoflavones from *P. lobata* extracts.

Isoflavones	Concentration (%, w/w)
Puerarin	7.2
Daidzin	3.8
Genistin	1.5
Total isoflavones	12.5

### 2.2. Effect on Infarct Volume in MCAo Rats

**Figure 2:**
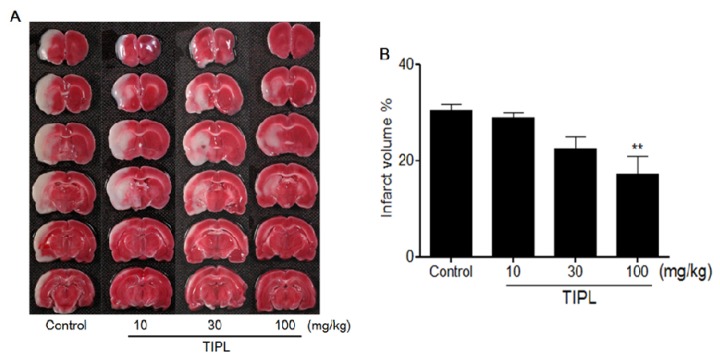
Anti-inflammatory effects of TIPL against ischemic brain injury. The dark pink area indicates the normal area, whereas the white area indicates the infarct area (**A**), and percentage of brain infarct area (**B**). All data are mean ± SD values (n = 10 per group). ** *p* < 0.01, significantly difference from the control group.

The white area in Figure 2A represents the infarction region in these sections. At 2 days after MCAo, the infarction regions were extended from the caudoputamen, parietal cortex and temporal cortex to the penumbral region (30.6% ± 2.5%). These effects were significantly reduced in the TIPL 100 mg/kg treated group at 0 and 2 h after MCAo to 16.9% ± 2.4% (*p* < 0.01) compared with the control group (Figure 2B).

### 2.3. Effects on COX-2, GFAP and OX-42 Expression

**Figure 3:**
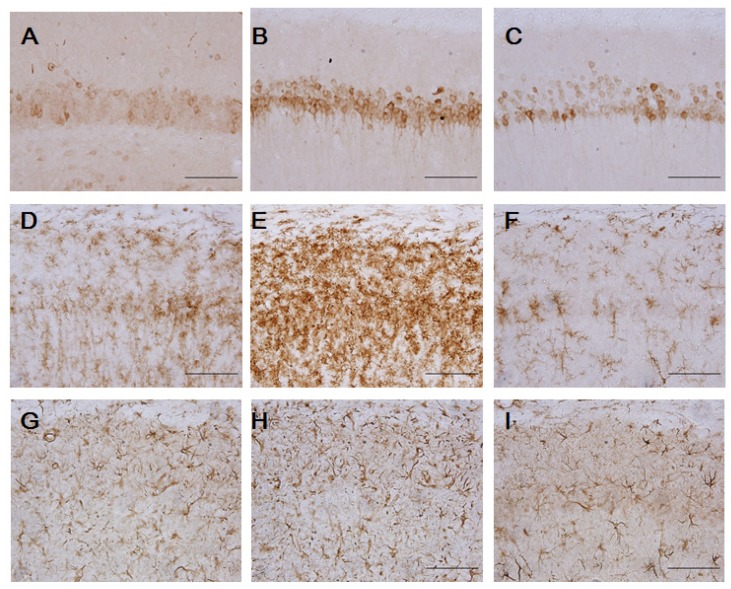
Inhibitory effect of TIPL on COX-2 (**A**,**B**,**C**), OX-42 (**D**,**E**,**F**), and GFAP (**G**,**H**,**I**) expression at 2 or 7 days after ischemia. Sham-operated group (A,D,G), control group (B,E,H), or TIPL 100 mg/kg treated group (C,F,I). Scale bar = 100 μm.

We evaluated immunoreactivity for COX-2 which is involved in the mechanisms of neurotoxicity associated within inflammation. Expression of COX-2 significantly increased in ischemic brain 2 days after MCAo, which was markedly inhibited by TIPL 100 mg/kg (Figure 3C). At 7 days after ischemia, we performed immunohistochemical staining of GFAP and OX-42 in rat brain slices to investigate whether TIPL 100 mg/kg has an effect on the inhibition of CA1 astrocytes and microglia activation. With OX-42 as a marker, no microglial cells were found in the sham-operated group (Figure 3D) and ischemia caused recruitment of microglial cells, which were especially clustered in the CA1 area with dying neurons (Figure 3E). However, TIPL 100 mg/kg markedly reduced this activated microglia (Figure 3F). Immunohistochemical staining with GFAP showed only few GFAP-positive astrocytes in the sham-operated groups (Figure 3G). However, ischemia induced an increase in GFAP-positive astrocytes, with hyperplasia and hypertrophy flanking the pyramidal neurons around hippocampal CA1 (Figure 3H). Ischemic that was treated with TIPL 100 mg/kg showed a marked decrease in reactive astrocytes compared with the ischemic group (Figure 3I). In short, TIPL attenuated ischemia induced COX-2 up-regulation, and ischemia-induced astrocyte and microglial activation in hippocampal CA1 region.

### 2.4. Discussion

In the present study, total isoflavones from *Pueraria* lobata extracts (TIPL) was demonstrated to have anti-inflammatory effects in *in vivo* ischemia. TIPL (100 mg/kg) reduced the brain infarct volume and attenuated ischemia-induced COX-2 up-regulation at 2 days after MCAo in rats. Moreover, TIPL reduced activation of GFAP and OX-42 at 7 days after MCAo in hippocampal CA1 region.

In focal cerebral ischemia, brain damage is commonly divided by the ischemic core and penumbra [11]. The nature of cell death in the ischemic core is known to be necrotic, whereas that in the penumbra is apoptotic [12]. In the ischemic core, the only way to recover cells is recanalization within 3 h after the onset. On the other hand, apoptotic cell death in the penumbra is induced by a biochemical cascade of events and can be protected by neuroprotective agents [1]. In MCAo, the ischemic core region consists of the lateral portion of the caudate putamen and parietal somato-sensory cortex [9]. TIPL 100 mg/kg treated group reduced brain damage, and the infarct area was restricted to part of the parietal cortex and caudate putamen, which is reported to be the ischemic core [10]. Therefore, the results indicate that TIPL can protect against neuronal damage in the penumbra region in a stroke *in vivo* model.

It has been reported that MCAo in rats can cause a severe and acute damage of neurons and oligodendrocytes in the ipsilateral hippocampal CA1 sector [13]. We also observed that MCAo increased ischemia-induced activation of COX-2, GFAP and OX-42 in the hippocampal CA1 regions. COX-2 is rate-limiting enzyme involved in arachidonic acid metabolism, thereby generating prostaglandins and thromboxanes, molecules that play important roles in inflammatory reaction [14] and reported to up-regulate in CA1 hippocampal cells until 2 days after ischemia [15]. An inflammatory reaction is known to be one of the major pathological mechanisms in focal cerebral ischemia [16]. Thus, treatments aimed at inhibiting COX-2 are viable strategies for targeting the late stages of ischemic injury [17]. In this study, TIPL inhibited COX-2 up-regulation at 2 days after ischemia in hippocampal CA1 region. Our results suggest that anti-inflammatory effects of TIPL after focal cerebral ischemia might be attributable to interrupting inflammatory reaction by the inhibition of COX-2 expression.

Focal cerebral ischemia is accompanied by reactive astrogliosis and activation of microglial cells in the hippocampal area [18]. Marked reactive gliosis was present in the entire MCA territory 7 days after MCAo [19]. Reactive gliosis can produce excess amounts of cytokines as well as inflammatory products that exacerbate ischemic damage [20]. Several studies have suggested that inhibiting glial activation attenuates ischemic injury [21,22]. In this study, are marked increase in GFAP and OX-42 immunoreactivity were observed in control group in comparison to sham-operated group while marked reduction were observed in TIPL 100 mg/kg treated group after MCAo.

There was increase in COX-2 expression at 2 days after MCAo, and microglia and astrocytes activations at 7 days after MCAo, which suggests that these inflammatory responses were caused by MCAo and that they in turn cause neuronal cell death in CA1 region. TIPL reduced the expressions of COX-2, microglia and astrocytes, which is consistent with previous studies demonstrating protection against ischemic brain damage by targeting inflammatory response also show reductions in glial activation [23,24,25].

Xu *et al.* reported that puerarin isolated from the dried root of *P.* *lobata* has neuroprotective effects against cerebral ischemia associated with an anti-apoptosis action [26]. A phenolic compound from *P. lobata* has inhibited beta-amyloid peptide (Aβ) toxicity in PC12 cells via a PI3K-dependent signaling pathway [27,28]. Also, a beneficial influence of some phytoestrogens in global cerebral ischemia has been previously reported. Genistein [29], catechin [30] or green tea extracts rich in phytoestrogens [31] have been shown to limit brain injury in the gerbil model of global cerebral ischemia. From above reports, the anti-inflammatory effects of TIPL against ischemic brain injury to be related to high contents of the isoflavones such as puerarin.

## 3. Experimental

### 3.1. Preparation of Total Isoflavones from P. lobata Extracts

*P. lobata* (300 g) was extracted with 70% ethanol (3,000 mL) for 3 h at 80 °C in a reflux apparatus. The extracts were filtered and concentrated under reduced pressure, and samples were lyophilized to yield a dark yellow powder. The yield of *P. lobata* extracts was 22.8%. The compositional analysis of total isoflavones from *P. lobata* extracts was determined on a high performance liquid chromatography (HPLC) system equipped with a Waters 1525 pump, a 2707 auto sampler and a 2998 photodiode array (PDA) detector. The chromatic separation was achieved at 30 °C on Waters Sunfire™ C18 (250 mm × 4 mm i.d., 5 μm particle size) column. The run time was set at 30 min and the flow rate was 1.0 mL/min and the sample injection volume was 10 μL. The mobile phase was 0.1% (v/v) formic acid (A)—100% acetonitrile (B) filtered through a 0.45 μm filter and degassed prior to use. Separation was achieved with gradient elution using 0.1% formic acid as a solvent. The gradient was reduced by 90% from 0 to 10 min, 75% from 10 to 15 min and 50% from 15 to 20 min, and was increased by 90% from 20 to 28 min to equilibrate the column. The flow rate was set at 1.0 mL/min, and samples were detected at 254 nm (Figure 1). After purification, puerarin (7.2%) was the major compound in the extracts, which also contained daidzin (3.8%) and genistin (1.5%). The dose of Total Isoflavones from *P. lobata* extracts (TIPL) for oral administration was calculated based on its isoflavone contents.

### 3.2. Animals and Treatments

Male Sprague-Dawley (SD) rats (180–200 g) were purchased from Samtako (Gyeonggi-do, Korea). Animals were housed at two rats per cage in an air-conditioned room at 23 ± 1 °C, 55%–60% relative humidity, and a 12 h light/dark cycle (07:00 lights on, 19:00 lights off), and were given a laboratory regular rodent diet. All animal experiments were carried out according to the guidelines of the Korea Food Research Institutional Animal Care and Use Committee. Focal ischemia/reperfusion was produced by a modification of the monofilament method described by Longa *et al.* [9]. Briefly, male SD rats undergoing middle cerebral artery occlusion (MCAo) were fasted overnight and deeply anesthetized by inhalation of isoflurane. The external carotid artery (ECA) was ligated and then cut just proximal to the external carotid bifurcation. The common carotid artery (CCA) and internal carotid artery (ICA) were temporarily occluded with a micro-vascular clip. A 4-0 nylon monofilament (0.36 mm in diameter), coated with silicone rubber, was introduced into the ECA. Correct placement of the suture was established when the suture was inserted at least 18 mm from the CCA/ICA bifurcation. At 2 h after MCAo, the suture was withdrawn to allow reperfusion. Throughout the experiment, the body temperature was monitored and maintained at 37 ± 0.5 °C with a homeothermic blanket system (Harvard Apparatus, Holliston, MA, USA). The sample treated groups were administrated TIPL (10, 30 and 100 mg/kg, *p.o.*) twice at 0 and 2 h after ischemia.

### 3.3. Tissue Preparation

To measure infarct volume, the rats were sacrificed 2 days after ischemia. The brains were removed quickly and cut into six coronal sections of 2 mm thickness. Sections were stained with 2% TTC (Sigma, St. Louis, MO, USA) in saline for 30 min at 37 °C. For the immunohistochemistry, rats were anesthetized, and brains were fixed by perfusion with 4% paraformaldehyde (PFA) after a transcardial wash-out with heparinized 0.5% sodium nitrite saline at 2 or 7 days after ischemia. The brains were removed and cut into 30 μm thick sections using a cryostat cryocut microtome (CM3050S; Leica, Heidelberg, Germany).

### 3.4. Measurement of Infarct Volume

TTC-stained sections were photographed and analyzed by a computerized image analysis system (Optimas, Media Cybernetics, Seattle, WA, USA) for measurement of infarct volume. The correlated infarct volume (mm^3^) was calculated by subtracting the intact volume of the ipsilateral hemisphere from the total volume of the contralateral hemisphere (mm^3^). The infarct volume (%) was calculated by dividing the correlated infarct volume (mm^3^) by the total volume (mm^3^) of the contralateral hemisphere.

### 3.5. Immunohistochemistry

Free-floating, 40 μm sections were reacted with a goat polyclonal antibody against COX-2 (1:100; Santa Cruz Biotechnology Inc., Santa Cruz, CA, USA), a mouse polyclonal antibody against OX-42 (1:100, Serotec, Oxford, UK), or a rabbit polyclonal antibody against GFAP (1:500, Sigma) overnight at room temperature. After incubation, the sections were reacted with anti-goat antibody, anti-mouse antibody or anti-rabbit antibody (1:200, Vector Laboratories, Burlingame, CA, USA) for 60 min, respectively and then reacted with an avidin–biotin–peroxidase complex kit (Elite ABC kit; 1:50, Vector Laboratories) at room temperature for 60 min. The avidin—biotin complex was visualized with 0.05% 3, 3-diaminobenzidine (DAB; Sigma) and 0.02% H_2_O_2_.

### 3.6. Statistical Analysis

All data were presented as the mean ± standard deviation (SD). The effects of different treatments were compared by one-way ANOVA followed by the Tukey’s post-hoc test using GraphPad Prism 4 (GraphPad Software Inc., La Jolla, CA, USA). *p* < 0.05 was considered statistically significant.

## 4. Conclusions

In our results showed that in a rat stroke model, TIPL protected the brain. Regarding the immunohistochemical study, the effect of TIPL may be attributable to its anti-inflammatory properties by the inhibition of COX-2 expression, microglia and astrocyte expression.
